# Optimization of the game improvement and data analysis model for the early childhood education major via deep learning

**DOI:** 10.1038/s41598-023-46060-9

**Published:** 2023-11-20

**Authors:** Yu Zhao, WenWen Gao, ShanShan Ku

**Affiliations:** 1 Department of Preschool Education, Nanyang Vocational College of Agriculture, Nanyang City, 473000 China; 2 Department of Command Tactics, Henan Police College, ZhengZhou City, 450000 China; 3 Department of Agricultural Engineering, Nanyang Vocational College of Agriculture, Nanyang City, 473000 China

**Keywords:** Computational science, Information technology, Scientific data

## Abstract

An ever-growing portion of the economy is dedicated to the field of education, intensifying the urgency of identifying strategies to secure the sector’s enduring prosperity and elevate educational standards universally. This study introduces a model for enhancing games and optimizing data analysis within the context of early childhood education (ECE) majors, hinging on deep learning (DL). This approach aims to enhance the quality of instruction provided to ECE majors and refine the effectiveness of their professional pursuits. This study commences by examining the incorporation of DL technologies within the domain of ECE and delving into their fundamental underpinnings. Subsequently, it expounds upon the design philosophy underpinning ECE games operating within the framework of DL. Finally, it outlines the game improvement and data analysis (GIADA) model tailored to ECE majors. This model is constructed upon DL technology and further refined through the integration of convolutional neural networks (CNN). Empirical findings corroborate that the DL-CNN GIADA model achieves data analysis accuracy ranging from 83 to 93% across four datasets, underscoring the pronounced optimization prowess bestowed by CNN within the DL-based GIADA model. This study stands as an invaluable reference for the application and evolution of artificial intelligence technology within the realm of education, thereby contributing substantively to the broader landscape of educational advancement.

## Introduction

The application of artificial intelligence (AI) technology to enhance the existing educational framework has emerged as the predominant approach in modern society for advancing the sustainable evolution of the education sector^1^. The domain of AI is presently experiencing rapid advancements in science and technology. This field is primarily concerned with programming computers to emulate higher-order cognitive processes and actions, encompassing the realization of computer-based intelligence and the development of computing systems replicating human brain-like intelligence^2^. Consequently, AI technology has become an indispensable tool across various industries within contemporary society. Deep learning (DL) technology, a pivotal facet of AI, has garnered significant attention due to its capacity to optimize the operational mechanisms of the education sector. This function has positioned it as a prominent area of research in contemporary society, with profound implications for the enhancement of early childhood education (ECE) programs through DL technology^3^. A substantial body of scholarly research substantiates this viewpoint.

Kevser^4^ emphasized the distinctive advantages of DL over conventional machine learning models, attributing them to the utilization of multi-layer neural networks capable of establishing end-to-end non-linear mappings from inputs to outputs. The recent strides in the application of AI to diverse gaming scenarios can be traced back to the successful integration of DL techniques, primarily aimed at addressing the challenges associated with strategy evaluation and optimization within the domain of reinforcement learning (RL)^4^. Hu et al.^5^ delineated a game-immersive teaching approach comprising four integral components: a revisit to foundational concepts, the deconstruction of complex subject matter into manageable segments, the formulation of stimulating activities fostering creativity, and a culminating phase designed to leave a lasting impression. Immersive learning via gaming serves as a catalyst for experiential learning, engendering a transformation from surface-level comprehension to profound understanding^5^. Lin and Yang^6^ introduced an innovative framework for designing, producing, and utilizing educational games inspired by DL and knowledge visualization. They further illustrated this framework through practical instances of game development. This model effectively mitigated the intricacies associated with game-based instruction and mitigated potential adverse consequences of gaming, thereby bridging the gap between educational game theory and practical implementation^6^. Aslam et al.^7^ underscored the evolving landscape of ECE prompted by ongoing education system reforms. Departing from conventional uniform approaches, ECE now prioritizes nuanced pedagogical practices. DL integration into kindergarten education aims to cultivate advanced reasoning and practical problem-solving skills in young learners. This approach incorporates DL into educational games, fostering rich and engaging problem-solving scenarios, promoting independent exploration, and stimulating creativity grounded in acquired knowledge^7^. Haritha et al.^8^ highlighted the surge in interest surrounding DL within the realm of education, underpinned by the global consensus on nurturing talents endowed with superior learning, problem-solving, and higher-order thinking abilities. Educational games, as fundamental activities in ECE, align seamlessly with the principles of DL. The attributes of curiosity, freedom, and openness exhibited by young learners complement the incorporation of DL, bestowing educational games with lasting educational value and significance^8^. Das et al.^9^ presented a novel approach to training a DL model for generating text within word games, dynamically altering and enhancing the in-game text based on pre-existing content. They developed Python code to simulate this text generation model and subsequently conducted comparative evaluations between the generated text and the original content. Their findings substantiated the effectiveness of the proposed method, particularly in the context of self-designed games^9^. The preceding research highlights the substantial advantages of employing DL within the gaming domain, particularly concerning strategy evaluation and optimization. Moreover, leveraging immersive learning experiences in gaming can facilitate experiential learning, elevating superficial comprehension to a deeper level of understanding. Nonetheless, extant research in this area lacks concrete examples and current scholarly support. Hence, this study’s primary objective is to substantiate the practical applications and advancements of DL in game-based education. It aims to achieve this goal through case studies and a review of recent research, addressing existing research gaps. Consequently, this study provides comprehensive theoretical guidance for effectively integrating DL into early childhood game-based education. Moreover, it explores the potential of DL in nurturing students’ creativity, critical thinking, and problem-solving abilities.

In conclusion, while the current strategy of harnessing DL technology for game design has made significant strides, its application within the realm of education warrants further exploration and elaboration. Promoting the integration and advancement of DL technology in the education sector is imperative. Initially, this study introduces the conceptual framework of employing DL technology in ECE. Subsequently, it delves into the foundational principles of game design in ECE. Ultimately, it presents a comprehensive game improvement and data analysis (GIADA) model tailored to the ECE domain, enriched by incorporating convolutional neural network (CNN) technology. This study holds the potential to significantly benefit the education sector at large by serving as a technical reference for the proliferation of both DL technology and ECE.

## ECE under DL

### Application of DL technology in ECE

#### Design concept

In the contemporary era, the extensive utilization of AI across diverse domains reflects the remarkable progress achieved in the realm of science and technology. Present-day societal expectations for ECE underscore the significance of two key principles: prioritizing children’s direct perception, practice, and experiential learning and emphasizing educational activities encompassing active exploration, cooperation, communication, and practical engagement. These principles harmonize seamlessly with the fundamental tenets of DL, which prioritize learning tailored to individual needs, foster critical comprehension, stimulate active creativity, and emphasize the transfer and application of knowledge. It is evident that DL stands as a potent tool for nurturing children’s development and fostering their overall well-being^10^. The study of DL holds paramount importance from both a theoretical and practical perspective, especially in light of recent educational reforms and pedagogical advancements that place a strong emphasis on foundational literacy skills. In the context of ongoing educational reforms and the evolving landscape of education, one of the foremost challenges is the imperative to cultivate proficiency in DL and the capacity to engage deeply with educational content^11^.

DL embodies key attributes encompassing active learning, critical thinking, and constructive learning within the learner’s educational journey. The pedagogical application of DL underscores the importance of socialization, emphasizing the seamless transfer of knowledge to real-world situations, cultivating learners’ innovative problem-solving acumen, and reinforcing their foundational knowledge base. The terminology “Deep Learning” initially emerged within the domain of AI, and today, DL, along with machine learning and AI technologies, has become indispensable in shaping the design and functionality of numerous intelligent products^12^.

DL exhibits a distinctive set of characteristics:

1. DL places a strong emphasis on higher-order thinking skills. Learners are encouraged to adopt an inquisitive mindset and engage in critical thinking throughout their educational journey. Instead of passively accepting information, they are urged to seek comprehension, assimilate knowledge, and apply it actively. Critical thinking equips students with the ability to approach new information critically, question it, conduct thorough analyses of existing data, explore the underlying structure of concepts, and contribute their interpretations and ideas. This approach fosters students’ capacity for independent exploration, creativity, and problem-solving^13^
2. Information integration is a central focus of DL. It involves the amalgamation of both new and existing knowledge, as well as the establishment of connections between them to construct new cognitive frameworks. These processes contribute to long-term memory retention and a profound understanding of complex subjects. The concept of information integration within DL underscores learners’ autonomy in acquiring knowledge at varying depths. Learners are encouraged to synthesize disparate pieces of information and formulate their unique frameworks for comprehending the world. Consequently, scrutinizing and examining newly acquired knowledge become essential to generate original ideas^14^
3. DL actively promotes learners’ critical construction of knowledge. Learners are encouraged to employ critical thinking skills when encountering new information. They critically assess newly acquired knowledge, discern the relationships between different pieces of information, integrate new knowledge into their existing cognitive structures, and construct new cognitive frameworks. In DL, learners take center stage, and this active engagement leads to the construction of new knowledge structures and a deeper understanding of essential knowledge components. Instructors play a supportive role, guiding students in bridging the gap between old and new information. The process of knowledge construction is integral to nurturing critical thinkers^15^
4. DL is fundamentally oriented toward knowledge transfer and real-world problem-solving. Rather than relying on rote memorization and superficial learning, DL encourages learners to think critically about how they can apply their acquired knowledge to solve novel problems. Teachers actively encourage students to question, investigate, and independently find solutions during their educational journey. This approach equips students with the skills needed to apply their knowledge in diverse contexts and fosters a culture of continuous innovation^16^.

To summarize, there is significant research potential in customizing and optimizing CNNs to enhance engagement, learning outcomes, and cognitive development within ECE games. Adapting CNN model architectures, including shallow networks and attention mechanisms, to align with preschool learners’ cognitive characteristics and requirements can result in improved model performance. Key steps involve comprehensive data collection, annotation, data augmentation, and preprocessing to ensure the diversity and quality of training data. Additionally, introducing personalized and adaptive elements that respond to the individual characteristics and performance of preschool learners can cater to diverse needs effectively. Real-time feedback and adaptive adjustments further enable the delivery of personalized learning experiences, promoting early childhood learning and cognitive development. This area of research holds immense promise for enhancing educational outcomes and providing personalized support within ECE games. Nonetheless, it warrants further investigation to address challenges such as data privacy and ethical concerns while ensuring preschoolers’ safety and healthy development within gaming environments.

#### Game optimization and data analysis

Within the realm of game optimization, several pivotal factors and metrics can be harnessed to enhance player experience, game equilibrium, and overall gaming performance. First and foremost, prioritizing player experience stands as a central objective in game optimization. A favorable player experience serves as a magnet to attract and retain player interest. This involves ensuring that game interfaces and user interface design are intuitive and user-friendly, thereby offering a seamless operational experience. Moreover, game difficulty and the learning curve should be meticulously balanced to enable players to progressively master game rules and skills, thereby providing a sense of accomplishment and challenge as they traverse from simplicity to complexity. Additionally, high-quality game audio and visual effects can augment immersion, immersing players in a more realistic and engaging experience. Lastly, a compelling game story and narrative can further immerse players within the game world, intensifying emotional resonance. Secondly, achieving game balance is of paramount importance. Game balance entails the equitable assessment and design of various elements intrinsic to the game, including characters, units, skills, and equipment. This signifies that choices and conflicts within the game possess significance, with no elements being excessively dominant or weak. The balance between characters or units must be harmonious, maintaining equilibrium between offense and defense, as well as ensuring a reasonable balance between skills and equipment. Sound balance design underpins fairness, diversity, and enjoyment within the gaming experience. Furthermore, overall game performance constitutes a critical factor necessitating optimization. Exceptional game performance guarantees a seamless gaming experience, averting lag, delays, and crashes. Game developers must be attentive to game speed and fluidity, ensuring the game can operate at a high frame rate across various devices. Effective resource management, encompassing the judicious allocation of memory and other resources, is vital to forestall resource depletion and performance deterioration. In the context of online multiplayer games, maintaining a robust network connection and delivering stellar multiplayer game performance assumes pivotal importance. Additionally, the timely resolution of errors and bug fixes stands as a pivotal facet of the game optimization process, ensuring game stability and sustained playability. In conclusion, game optimization enhances player experience, upholds game balance, and optimizes overall game performance. By steadfastly addressing these pivotal factors, game developers can furnish players with an improved gaming experience, preserve equilibrium within the game, and perpetually elevate the overall gaming performance. This approach attracts a broader player base and furnishes them with a more enjoyable and gratifying gaming encounter.

Data analysis assumes a pivotal role in the realm of game design, affording developers profound insights through the scrutiny of player behavior, in-game interactions, and performance metrics. Player behavior analysis, in particular, unveils the decisions players make within the game, tasks they successfully accomplish, and their interactions with fellow players. This trove of data unveils player preferences, gaming routines, and individual play styles, thereby equipping developers with a richer understanding of player needs and enabling the delivery of gaming experiences that resonate with player expectations. Complementing this, the analysis of player feedback assumes another vital dimension. By delving into player opinions, suggestions, and comments, developers can gauge player contentment, inclinations, and areas of discontent within the game. Qualitative feedback data empowers developers to fine-tune the game design, address pain points, and culminate in an elevated player experience. A/B testing represents a frequently deployed experimental methodology wherein developers juxtapose different game designs, features, or versions to discern which alterations yield superior outcomes. Through the analysis of behavior and feedback from distinct player groups, developers can pinpoint player preferences for varying design elements and subsequently implement adjustments and enhancements. Further enhancing these insights, group analysis involves the segmentation of players grounded in data, facilitating comprehension of the conduct and performance of discrete player cohorts. By juxtaposing metrics derived from diverse player segments, developers glean insights into the requisites and inclinations of distinct player groups, thereby enabling the delivery of more tailored game content and services. In conclusion, the arsenal of data analysis constitutes a potent instrument of paramount import within the ambit of game design. By harnessing data analysis, developers glean profound insights into player behavior and predilections, identify trends and areas necessitating refinement within game design, and persistently fine-tune and augment the gaming experience to cater to the needs and anticipations of players.

#### Game mechanics

Incorporating machine learning and AI algorithms into the data analysis process can automate decision-making, personalize gaming experiences, and dynamically optimize game mechanisms. Primarily, these algorithms streamline the process of analyzing vast datasets, efficiently automating decision-making. Through model training, algorithms can automatically detect and predict pertinent patterns and trends, offering real-time insights and suggesting strategies. For example, automated user segmentation and behavioral analysis can provide personalized game content and recommendations or save states tailored to individual interests and behavioral patterns. Furthermore, machine learning and AI algorithms empower game developers to create personalized gaming experiences. Developers gain insights into each player’s preferences, inclinations, and behavioral patterns by analyzing player data and leveraging recommendation systems and personalized modeling techniques. This knowledge informs the delivery of personalized game content, missions, challenges, and rewards, resulting in heightened player engagement, satisfaction, and ultimately, improved player retention rates and user loyalty. Moreover, machine learning and AI algorithms facilitate the dynamic optimization of game mechanisms. These algorithms analyze player behavior and feedback data, identifying issues, addressing imbalances, and refining in-game requirements. Developers can utilize these insights to fine-tune game difficulty, rectify errors, enhance game flow, and improve game quality effectively. However, integrating machine learning and AI algorithms in game development poses certain challenges. Firstly, substantial quantities of high-quality data are requisite for model training, and acquiring an adequate volume of suitable data can sometimes prove challenging. Additionally, algorithms’ training and iteration process may necessitate significant computational resources and time, and ensuring data privacy and security remains a paramount concern. Nevertheless, the adoption of machine learning and AI algorithms in game development offers myriad advantages. These technologies empower developers with a deeper understanding of player needs and behavioral patterns, enabling the provision of personalized and adaptive gaming experiences that enhance player engagement and satisfaction. Furthermore, these algorithms provide real-time decision support, optimize game mechanisms and processes, and facilitate intricate game design and operational strategies. Their application elevates game quality, augments user retention, and generates commercial value.

### Design concept of ECE games based on DL

**Figure 1:**
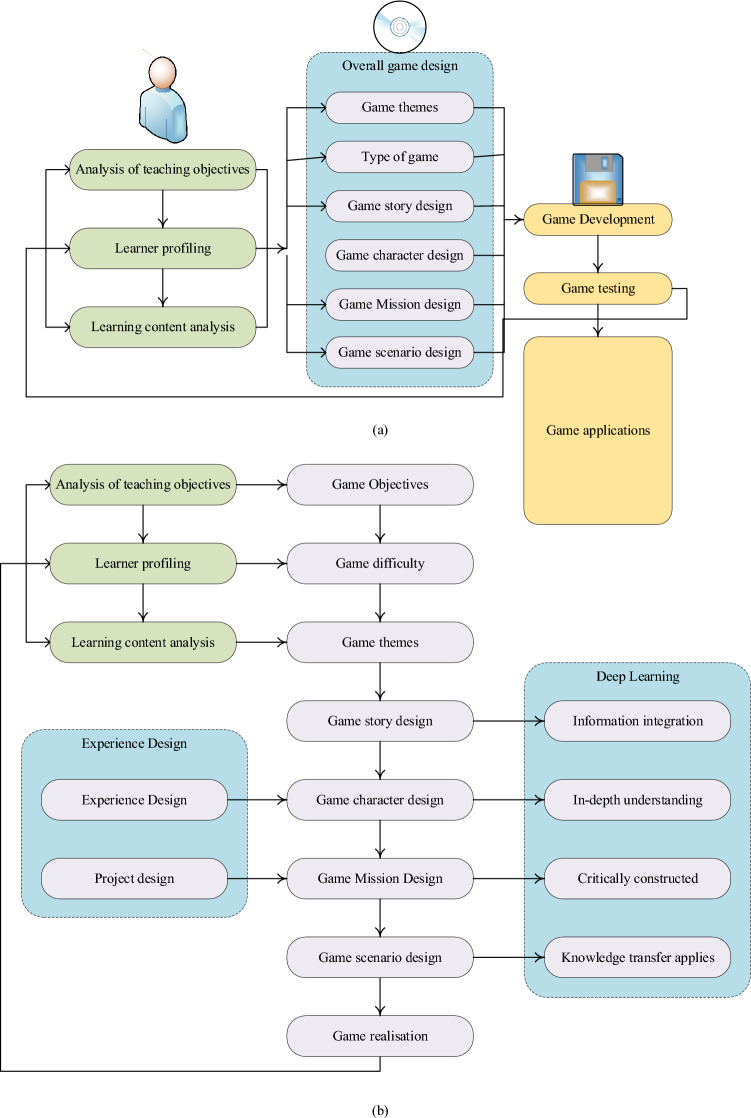
Design concept of DL-based ECE games (**a** the design concept of ECE games; **b** the game design concept under DL).

The creation and advancement of educational games constitute an extensive endeavor that necessitates proficiency across various domains. This comprehensive project relies on the collaborative efforts of game strategists, program architects, character designers, art engineers, music producers, educators, and instructors to craft educational content effectively. The typical design framework for educational games encompasses elements such as the scrutiny of educational objectives, learner profiles, learning materials, overarching game design, game production, quality assessment, and game implementation^17^. Figure 1 illustrates the design concept of ECE games leveraging the principles of DL.

As depicted in Fig. 1, game design within the realm of the education industry exhibits several distinctive characteristics: ① Independent learning: Educational games foster independent learning as players assume the roles of game protagonists and navigate a series of challenges. Engaging and enjoyable gameplay encourages active player involvement, leading to a higher likelihood of profound learning experiences^18^. ② Adaptive design: The design of instructional games accommodates varying student skill levels. Learners are attracted to games with captivating settings, well-developed characters, and a range of challenge levels. Players have the flexibility to tailor their experiences by choosing character types and challenge levels within a game, promoting DL. Consequently, instructional games provide an environment conducive to DL^19^. ③ Immersive engagement: Meticulously crafted game characters, captivating visuals, and thoughtfully designed game tasks captivate learners, immersing them in the gaming experience. Problem-solving serves as a prerequisite for completing these tasks. Therefore, in the creation of educational games, designers must align gaming activities with learning objectives, techniques, and content. The specific design should enable learners to complete tasks while acquiring knowledge and applying it to real-world issues, a hallmark of DL. Consequently, educational games inspire learners to delve into DL experiences^20^.

Furthermore, the ECE game model developed through DL technology should adhere to the following guiding principles: ① Learner-centered approach: Educational games must be designed with a strong focus on learners, aiming to enhance their learning experiences and acknowledging their central role. Design should prioritize student engagement by selecting learning content that aligns with their interests, addresses societal needs, and fosters improved academic performance^21^. ② Integration of game and teaching design: The process of creating instructional games is intricate, requiring collaboration among diverse experts. In terms of instructional design, educators must define learning goals and content, considering DL characteristics, learner analysis, and other factors. They should collaborate closely with game designers to align the game’s theme with educational objectives and content^22^. Game designers are responsible for planning elements such as characters, missions, narratives, rules, items, and systems, while artists enhance game scenes, characters, and special effects^23^. Game producers implement these elements within the game’s flow. This necessitates a seamless integration of game and instructional design^24^. ③ Emphasis on innovation: Game innovation is integral to the game’s quality and appeal. Novelty plays a pivotal role in attracting new players and defining a game’s excellence. Recognizing that every student possesses creative potential, the design of educational games should provide opportunities for students to exercise their creativity and actively encourage innovative thinking^25^.

Leveraging DL techniques for analyzing and simulating preschool children’s cognitive development using game data can yield invaluable insights. With their inherent capacity for automated learning and feature extraction, DL algorithms are well-suited to unveil concealed patterns and trends within vast data sets of game-related information. Such datasets may encompass various aspects of children’s interactions with the game, including behavioral patterns, response times, scores, progress, language expressions, facial expressions, and more. DL technology facilitates the extraction of specific features and behavioral patterns from game data. For instance, by scrutinizing children’s behavior in games, DL algorithms can shed light on their attention, concentration, and problem-solving skills. Time-series analysis enables tracking children’s learning curves and progress rates, helping identify potential learning bottlenecks or areas requiring improvement. The application of sentiment analysis techniques provides insights into children’s emotional states and game experiences, thereby aiding game design optimization and personalized instruction. DL algorithms empower the extraction of crucial features from game data, enabling the analysis of children’s learning progress and identifying potential areas for enhancement. For example, image analysis techniques can extract features from images, providing insights into how children perceive and process visual information. Natural language processing can analyze children’s language expressions during gameplay, offering insights into their comprehension of game content and emotional responses. These DL techniques exhibit superior predictive accuracy and representational capacity compared to traditional machine learning methods. They excel in handling complex data patterns and can learn more abstract and useful feature representations from raw data. In contrast to conventional machine learning methods, DL algorithms are particularly well-suited for processing large and high-dimensional game datasets. Nonetheless, DL algorithms also face certain challenges. They typically necessitate a substantial volume of training data, demanding high data quality standards. Additionally, both the training and inference processes of DL models require significant computational resources, potentially introducing efficiency concerns. In summary, applying DL techniques to game data holds promise for providing valuable insights into the cognitive development of preschool children. These insights can be instrumental in optimizing educational game design, personalizing instruction, and evaluating children’s learning progress. Nevertheless, the effective implementation of DL algorithms must address specific technical and computational challenges.

## ECE game design model under DL

This study highlights the effectiveness of employing transfer learning and fine-tuning techniques to train CNN models for enhanced performance on ECE game data. Pre-trained CNN architectures, having acquired general features from extensive image datasets, serve as an initial foundation. Fine-tuning then comes into play, integrating these general features with the unique characteristics of ECE game data to enhance the model’s adaptability to educational gaming tasks. The approach involves freezing certain network layers to retain general features while progressively unfreezing other layers, allowing the model to better tailor itself to specific tasks. Furthermore, data preprocessing and augmentation strategies contribute to increased data diversity and quality, ultimately enhancing the model’s generalization capabilities. In summary, the utilization of transfer learning and fine-tuning strategies proves highly effective in training CNN models with ECE game data, resulting in improved learning outcomes and adaptability to the unique characteristics of preschoolers. This approach holds the promise of delivering more personalized and high-quality educational gaming experiences for preschool-aged children. The real-time analysis and assessment of preschoolers’ interactions and learning progress during gameplay using CNNs can offer valuable insights to educators and parents, facilitating personalized learning support. Metrics and behavioral patterns that can be extracted through CNN analysis encompass behavior recognition, cognitive load assessment, learning progress tracking, and emotion recognition. These insights empower education professionals to better understand preschoolers’ engagement, attention, learning preferences, and emotional states, enabling targeted feedback and support tailored to their developmental needs. By closely monitoring performance and reactions, educators and parents can identify preschoolers’ strengths and challenges in various areas, enabling the provision of appropriate learning materials and guidance. This real-time analysis approach promotes personalized learning for preschoolers, enhancing their engagement, learning outcomes, and cognitive development. However, the implementation of this approach necessitates careful consideration and the development of solutions to ensure data security and privacy protection, as well as to mitigate overreliance on technological limitations. The field of machine learning revolves around the utilization of data and experience to enhance the efficiency of computer systems, enabling them to perform the same or similar tasks effectively in the future. Machine learning can typically be categorized into various types based on the nature of feedback signals, including supervised learning, semi-supervised learning, unsupervised learning, and RL. RL, in particular, is distinctive in that it operates without pre-existing samples. Instead, the RL agent acquires (state, action, reward) samples through interactions with its environment, enabling trial-and-error learning to continuously refine its approach in pursuit of maximizing cumulative rewards^26^. RL often employs the Markov Decision Process (MDP) as a mathematical model. An MDP can be formally represented as a five-tuple (**S**, **A**, **T**, **R**, **γ**): **S** represents the set of states; **A** denotes the set of actions; **T** signifies the probability of transitioning from the current state to a specific state upon executing an action; **R** corresponds to the associated reward; and **γ** represents the discount rate^27^. Typically, the symbol **π** is used to represent a policy:

1 $$ {\varvec{\pi}}\left( {{\mathbf{s}},{\mathbf{a}}} \right):{\mathbf{S}} \times {\mathbf{A}} \to \left[ {0,1} \right] $$

Equation (1) describes the probability associated with the execution of action **a** in state **s**^28^. Building upon this, RL aims to discover a strategy that maximizes the cumulative reward. Consequently, this study formulates the cumulative loss reward as depicted in Eq. (2).

2 $$ {\varvec{J}}\left( {\varvec{\pi}} \right) = {\varvec{E}}_{{{\varvec{s}}_{0} ,{\varvec{a}}_{0} , \ldots }} \left[ {\mathop \sum \limits_{{{\varvec{t}} = 0}}^{\infty } {\varvec{\gamma}}^{{\varvec{t}}} {\varvec{r}}\left( {{\varvec{s}}_{{\varvec{t}}} ,{\varvec{a}}_{{\varvec{t}}} } \right)} \right] $$

Similarly, the state-value function is defined as Eq. (3).

3 $$ {\varvec{V}}^{{\varvec{\pi}}} \left( {\varvec{s}} \right) = {\varvec{E}}\mathop \sum \limits_{{{\varvec{t}} = 0}}^{\infty } {\varvec{\gamma}}^{{\varvec{t}}} {\varvec{r}}\left( {{\varvec{s}}_{{\varvec{t}}} ,{\varvec{a}}_{{\varvec{t}}} } \right)\left| {{\varvec{s}}_{0} = {\varvec{s}},{\varvec{\pi}}} \right| $$

It can also be written as Eq. (4).

4 $$ {\varvec{Q}}^{{\varvec{\pi}}} \left( {{\varvec{s}},{\varvec{a}}} \right) = {\varvec{E}}\mathop \sum \limits_{{{\varvec{t}} = 0}}^{\infty } {\varvec{\gamma}}^{{\varvec{t}}} {\varvec{r}}\left( {{\varvec{s}}_{{\varvec{t}}} ,{\varvec{a}}_{{\varvec{t}}} } \right)\left| {{\varvec{s}}_{0} = {\varvec{s}},{\varvec{a}}_{0} = {\varvec{a}},{\varvec{\pi}}} \right| $$

In addition, this study uses two methods based on function and strategy. Equation (5) indicates the method based on function.

5 $$ {\varvec{Q}}^{{\varvec{\pi}}} \left( {{\varvec{s}},{\varvec{a}}} \right) = {\varvec{Q}}^{{\varvec{\pi}}} \left( {{\varvec{s}},{\varvec{a}}} \right) + {\varvec{\alpha}}\left( {{\varvec{r}} + \user2{\gamma maxQ}\left( {\user2{s^{\prime}},\user2{a^{\prime}}} \right) - {\varvec{Q}}\left( {{\varvec{s}},{\varvec{a}}} \right)} \right) $$

In Eq. (5), ***α*** represents the learning rate^29^. In scenarios where the state and action spaces are extensive or even continuous, direct computation of the value function for every state-action pair becomes unfeasible. Consequently, employing a methodology akin to supervised learning becomes imperative, wherein a model is utilized to approximate the value function. This can be expressed as follows:

6 $$ {\varvec{Q}}\left( {{\varvec{s}},{\varvec{a}}} \right) \approx {\varvec{Q}}\left( {{\varvec{s}},{\varvec{a}};{\varvec{\theta}}} \right) $$

The mean square error of the updated difference during the model learning process is calculated according to Eq. (7).

7 $$ {\varvec{L}}_{{\varvec{i}}} \left( {{\varvec{Q}}_{{\varvec{i}}} } \right) = {\varvec{E}}_{{{\varvec{s}},{\varvec{a}},{\varvec{r}},\user2{s^{\prime}}}} [\left( {{\varvec{r}} + \user2{\gamma maxQ}\left( {\user2{s^{\prime}},\user2{a^{\prime}}} \right) - {\varvec{Q}}\left( {{\varvec{s}},{\varvec{a}}} \right))^{2} } \right] $$

In addition, the policy-based method is expressed as Eq. (8).

8 $$ \nabla_{{\varvec{w}}} {\varvec{J}}\left( {\varvec{w}} \right) = {\varvec{E}}\left[ {\nabla_{{\varvec{w}}} {\varvec{log}}{{\varvec{\uppi}}}_{{\varvec{w}}} \left( {{\varvec{a}}\left| {\varvec{s}} \right|} \right){\varvec{Q}}^{{\varvec{\pi}}} {\varvec{w}}\left( {{\varvec{s}},{\varvec{a}}} \right)} \right] $$

Then, the parameter is updated via Eq. (9).

9 $$ {\varvec{w}} = {\varvec{w}} + {\varvec{\eta}}\nabla_{{\varvec{w}}} {\varvec{J}}\left( {\varvec{w}} \right) $$

**Figure 2:**
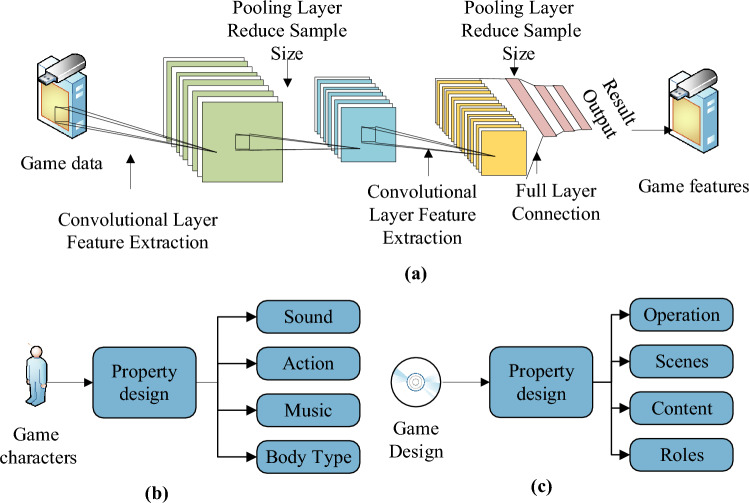
CNN-optimized GIADA model for the ECE major (**a** the overall design of the model; **b** the character design; **c** the overall design of the game).

Consequently, this study introduces a GIADA model specifically tailored for the ECE major, leveraging DL technology^30^. Furthermore, the GIADA model undergoes optimization through CNN technology to enhance its performance^31^. Figure 2 presents the CNN-optimized GIADA model.

Equation (10) indicates feature extraction of data by CNN.

10 $$ {\mathbf{H}}_{{\varvec{i}}} = {\varvec{f}}\left( {{\mathbf{W}}_{{{\varvec{i}} \otimes }} {\mathbf{H}}_{{{\varvec{i}} - 1}} + {\mathbf{b}}_{{\varvec{i}}} } \right) $$

In Eq. (10), ***i*** represents the network convolution level; **W** signifies the calculation weight; **b** refers to the offset vector in the calculation process. The activation function activates this offset vector to generate the feature vector **H**_***i***_. The pooling process of the CNN is articulated in Eq. (11).

11 $$ {\mathbf{H}}_{{\varvec{i}}} = {\varvec{subsampling}}\left( {{\mathbf{H}}_{{{\varvec{i}} - 1}} } \right) $$

Equation (12) illustrates the ultimate mapping outcome of altered features following multiple pooling. This outcome is further combined with representation and classification through the fully connected network.

12 $$ {\mathbf{Y}}\left( {\varvec{m}} \right) = {\varvec{P}}\left( {{\mathbf{L}} = {\varvec{l}}_{{\varvec{m}}} \left| {{\mathbf{H}}_{0} ;\left( {{\mathbf{W}},{\mathbf{b}}} \right)} \right.} \right) $$

Equation (12) introduces the variables used in the subsequent equations: ***m*** represents the index of the label category; **L** denotes the loss function; ***P*** signifies the mapping operation. The loss function is expressed as follows:

13 $$ {\mathbf{NLL}}\left( {{\mathbf{W}},{\mathbf{b}}} \right) = - \mathop \sum \limits_{{{\varvec{m}} = 1}}^{{\left| {\mathbf{Y}} \right|}} {\mathbf{logY}}\left( {\varvec{m}} \right) $$

14 $$ {\mathbf{MSE}}\left( {{\mathbf{W}},{\mathbf{b}}} \right) = \frac{1}{{\left| {\mathbf{Y}} \right|}}\mathop \sum \limits_{{{\varvec{m}} = 1}}^{{\left| {\mathbf{Y}} \right|}} ({\mathbf{Y}}\left( {\varvec{m}} \right) - {\hat{\mathbf{Y}}}\left( {\varvec{m}} \right))^{2} $$

A two-norm term is usually added to the final loss function to reduce the overfitting of the network parameters:

15 $$ {\varvec{E}}\left( {{\mathbf{W}},{\mathbf{b}}} \right) = {\mathbf{L}}\left( {{\mathbf{W}},{\mathbf{b}}} \right) + \frac{{\varvec{\lambda}}}{2}{\mathbf{W}}^{{\mathbf{T}}} {\mathbf{W}} $$

16 $$ {\mathbf{W}}_{{\varvec{i}}} = {\mathbf{W}}_{{\varvec{i}}} - {\varvec{\eta}}\frac{{\partial {\varvec{E}}\left( {{\mathbf{W}},{\mathbf{b}}} \right)}}{{\partial {\mathbf{W}}_{{\varvec{i}}} }} $$

17 $$ {\mathbf{b}}_{{\varvec{i}}} = {\mathbf{b}}_{{\varvec{i}}} - {\varvec{\eta}}\frac{{\partial {\varvec{E}}\left( {{\mathbf{W}},{\mathbf{b}}} \right)}}{{\partial {\mathbf{b}}_{{\varvec{i}}} }} $$

where ${\varvec{\eta}}$ denotes the learning rate^32^.

Building upon these principles, this study introduces the DL-CNN GIADA model and comprehensively evaluates its performance. Several DL architectures and algorithms are well-suited for optimizing model improvements in ECE games. However, the choice of a specific technology should be contingent upon the data characteristics and the objectives of the model:

1. CNN: CNNs are widely recognized for their effectiveness in tasks related to images. They possess the capability to learn and extract pertinent features from visual data autonomously. In the context of preschool education, CNNs can be employed to analyze visual elements or patterns within educational games
2. RNN: RNNs are particularly well-suited for modeling sequential data, such as behavioral or interaction sequences within educational games. RNNs excel at capturing temporal dependencies and acquiring representations that mirror the dynamic aspects of the gaming process
3. GAN: GANs offer valuable utility in generating new game scenarios or content tailored to the specific educational requirements of preschoolers. By simultaneously training both generator and discriminator networks, GANs facilitate the creation of entirely novel game elements that align with educational objectives.

In terms of predictive accuracy, DL architectures, particularly CNNs, have consistently demonstrated exceptional performance across various domains, including computer vision and natural language processing. Their automatic learning capability equips them with a notable advantage in handling intricate patterns and vast datasets, surpassing traditional machine learning methods in accuracy. However, it is essential to weigh this accuracy against the computational efficiency of DL algorithms. DL models typically feature a higher number of parameters and demand more computational resources during both training and inference phases. Consequently, this results in prolonged training times and higher hardware requirements than traditional machine learning models. Conventional machine learning techniques such as decision trees, support vector machines, and random forests often offer superior computational efficiency, especially for specific tasks and smaller datasets. They typically involve less data preprocessing, exhibit smaller model sizes, and boast faster training and deployment speeds. In summary, DL architectures such as CNNs, RNNs, and GANs exhibit substantial potential to optimize model enhancements in preschool education games. Their capacity to learn intricate patterns and features contributes to their ability to deliver highly accurate predictions. Nonetheless, it is imperative to strike a balance between the computational resources demanded by DL algorithms and the computational efficiency offered by traditional machine learning methods. Based on the above content, the model designed in this paper primarily consists of two key aspects:

1. Method: The deep learning convolutional neural network (DL-CNN) is a machine learning model with the capacity to learn and extract features from input data autonomously. It harnesses components, including convolutional layers, pooling layers, and fully connected layers, for the processing and analysis of data, facilitating the acquisition of intricate patterns and relationships
2. Structure:
  1. Input layer: This layer receives input data, which may encompass images, text, or other data types originating from educational games
  2. Convolutional layer: Utilizing convolution kernels (filters), this layer filters the input data to extract local features
  3. Pooling layer: Subsequently, the pooling layer subsamples the feature maps from the convolutional layer. This process reduces data dimensions while preserving essential feature information
  4. Fully connected layer: The output from the pooling layer is connected to one or more neural units within this layer. It conducts linear operations with connection weights and introduces non-linear characteristics through activation functions
  5. Output layer: Depending on the specific task requirements, the output layer can be a softmax layer for classification or a linear layer for regression. During the model training process, a loss function is employed to gauge the disparity between the model’s predictions and the actual labels. Optimization algorithms, such as gradient descent, are then utilized to update the model’s parameters, minimizing the loss function.

Utilizing DL models in ECE represents a promising technological advancement that holds the potential to provide personalized learning experiences for young children while offering valuable educational insights. However, the application of DL models in this context necessitates a delicate equilibrium between ethical considerations and privacy concerns. First and foremost, the paramount concern is safeguarding the data privacy of young children. When collecting and utilizing game data from young children, obtaining explicit consent from parents or guardians is imperative, and strict adherence to relevant data protection regulations is mandatory. The data collection process must be meticulously designed to minimize the acquisition of personal information, with robust security measures implemented to safeguard data storage and transmission. Secondly, transparency and interpretability play pivotal roles. DL models, often intricate and opaque, demand initiatives to enhance their transparency. This measure ensures that parents and educators can comprehend the model’s functioning and its impact on children’s learning, fostering trust and potentially increasing its acceptance. Furthermore, ethical considerations extend to educational fairness. The design and training of DL models should actively circumvent discriminatory assessments grounded in individual characteristics, steadfastly upholding the principles of fairness and equal opportunity. These models should concentrate on recognizing individual disparities while eschewing bias or disparate treatment. Strict adherence to data protection regulations is imperative throughout the optimization process while simultaneously providing effective personalized learning experiences for young learners. This can be achieved by anonymizing and aggregating young children’s data to preserve privacy. Secure and controlled mechanisms for data usage and sharing should be established. Moreover, transparent communication of personalized learning objectives to both parents and young children, accompanied by a consensus-building process, can contribute to enhancing the acceptability of educational approaches. In conclusion, the utilization of DL models to enhance ECE requires meticulous consideration of ethical concerns and privacy safeguards. Achieving a harmonious balance between optimizing the learning process and preserving the data privacy of young children is the key to advancing technology and offering effective personalized learning experiences. This, in turn, promotes cognitive development and improved learning outcomes for young children.

## Data design for ECE games

This study utilizes four datasets to enhance the model’s computational performance and bolster its labeling and classification capabilities. The datasets employed are as follows:

1. Video Game Sales with Ratings (VGSR) (https://www.kaggle.com/datasets/rush4ratio/video-game-sales-with-ratings) dataset: This dataset, available on Kaggle encompasses information regarding the sales and ratings of video games released from 1980 to 2017. It comprises 16,719 entries categorized into 14 distinct fields
2. Ten-year game data (https://www.kaggle.com/): Published on Kaggle, this dataset contains 18,624 data points distributed across ten fields
3. Amazon game dataset (https://cseweb.ucsd.edu/~jmcauley/datasets.html#amazon_reviews): This dataset represents the product segment of Amazon’s shopping interface. Amazon, a global e-commerce leader, ranked second in the global e-commerce standings in 2017, providing valuable analytical insights
4. Board-game-data dataset (https://www.kaggle.com/datasets/mrpantherson/board-game-data): With a sample size of 81,312 entries and 20 fields, this dataset is a valuable resource for analysis.

Table 1 provides an overview of the experimental environment settings.

**Table 1:** Experimental conditions.

Serial number	Software/hardware	Version/model
1	Processor	Intel(R) Core(TM) i7-3520 M CPU @ 2.90 GHz 2.90 GHz
2	Memory	4 GB
3	Operating system	Windows10 × 64
4	Java Development Kit	1.8

GIADA models in the realm of ECE confront formidable challenges and potential limitations when handling data and conducting analyses. Data quality emerges as a pivotal concern since the precision of raw data profoundly influences subsequent analyses and models. Issues such as noise, missing values, or erroneous data necessitate data cleaning and rectification. Furthermore, the substantial volume of data and high-dimensional features present complexities in terms of computational resources and model interpretability. In response to these challenges, contemporary GIADA models within ECE increasingly embrace DL algorithms. With its hierarchical neural network architecture and automatic feature extraction capabilities, DL excels in managing intricate data patterns and yields superior accuracy. A primary advantage of DL algorithms in data processing lies in their innate capacity to autonomously learn and extract valuable features, diminishing reliance on manual feature engineering. This streamlines model design and fine-tuning, enabling the handling of diverse and unstructured data types such as images, speech, and text. Moreover, DL algorithms augment data processing efficiency through parallel computing, optimization algorithms, and advancements in hardware infrastructure. The employment of parallel computing, particularly with graphics processing unit clusters, accelerates the processing and analysis of extensive data. Ongoing enhancements in optimization algorithms facilitate more effective searches for optimal parameter combinations and diminish computational resource demands via parameter compression and model acceleration techniques. These strides empower GIADA models in the realm of ECE. However, DL algorithms introduce potential limitations. The complexity of DL algorithm models and the expanded parameter count necessitates a relatively substantial volume of training data, which may be impeded by issues such as data acquisition and privacy safeguards. Furthermore, DL algorithms pose challenges in terms of model interpretability, as some models may be challenging to elucidate regarding the rationale behind their decisions. This aspect can present difficulties for educational professionals seeking to comprehend and apply them. In summary, GIADA models in ECE reap substantial benefits from the adept handling of intricate data patterns and heightened accuracy facilitated by DL algorithms. Nevertheless, challenges linked to data quality, computational complexity, and model interpretability must be systematically addressed. With the perpetual evolution of DL technology and the accumulation of application experience, substantial strides in data processing efficiency and precision are anticipated in the future.

## Evaluation of the DL-CNN GIADA model for the ECE major

### Evaluation of the DL-based GIADA model

**Figure 3:**
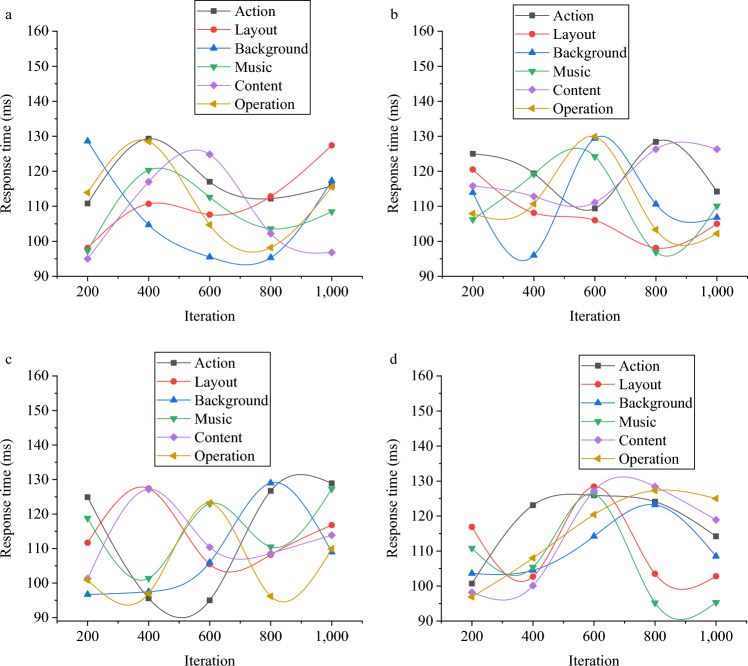
Performance evaluation of the DL-based GIADA model (**a** the VGSR dataset; **b** Ten Year dataset; **c** the Amazon dataset; **d** the board-game-data dataset).

This study assesses the DL-based GIADA model, with the aforementioned game datasets serving as the primary research focus, to comprehensively investigate its overall design and data analysis performance. The evaluation of the model encompasses six key aspects: game actions, layout, backgrounds, music, content, and operations. Figure 3 presents the holistic performance of the model in game design.

**Figure 4:**
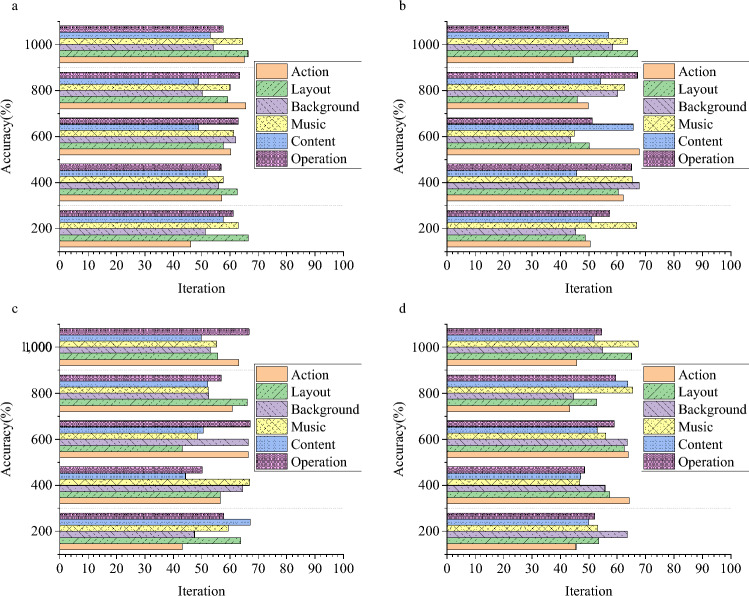
Data analysis performance of the DL-based GIADA model (**a** the VGSR dataset; **b** Ten Year dataset; **c** the Amazon dataset; **d** the board-game-data dataset).

As depicted in Fig. 3, the response times of the DL-based GIADA model across the four datasets fall within the range of approximately 95–130 ms, 97 –128 ms, 94–126 ms, and 90–126 ms, respectively. Furthermore, this study conducts an in-depth analysis of the model’s data analysis performance within the context of the games. Figure 4 provides a visual representation of the evaluation outcomes pertaining to the model’s data analysis performance.

As depicted in Fig. 4, the DL-based DIADA model attains accuracy scores ranging from 46 to 66% across the analysis of data related to six distinct aspects of a game: namely, action, layout, background, music, content, and operation. These figures indicate that the model’s data analysis performance falls below the desired standards. Consequently, this study employs a CNN model to optimize the model, with the aim of enhancing its performance. The evaluation outcomes of the optimized model are elaborated upon in the following section.

### Evaluation of the DL-CNN GIADA model

**Figure 5:**
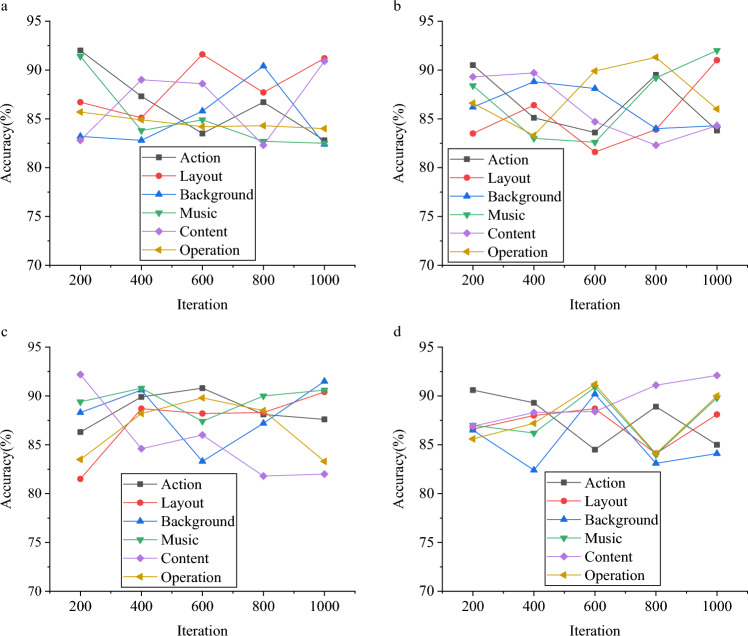
Performance evaluation of the DL-CNN GIADA model (**a** the VGSR dataset; **b** Ten Year dataset; **c** the Amazon dataset; d: the board-game-data dataset).

The GIADA model for the ECE major, employing DL technology, is further refined through integration with the CNN model, culminating in the DL-CNN GIADA model. Comprehensive evaluations are conducted to assess the model’s performance across a range of factors. Figure 5 presents the outcomes of these extensive performance evaluations of the DL-CNN GIADA model.

Figure 5 illustrates the remarkable performance of the DL-CNN GIADA model in both game design and data analysis for the ECE major. Notably, the data analysis accuracy of the DL-CNN GIADA model ranges from 83 to 93%, 82 to 93%, 81 to 93%, and 83 to 91%. These results underscore the substantial optimization impact of CNN technology on the DL-based model. Consequently, this study makes a substantial contribution to enhancing the game design model for the ECE major. In contrast to the study conducted by Meng et al.^33^, this study’s model presents substantial advantages and innovations. While Meng et al.’s research has indeed achieved commendable milestones in the domain of DL game optimization and data analysis, this paper’s model propels further enhancements and extensions in terms of granularity. It becomes apparent that, when juxtaposed with the conventional DL methods embraced by Meng et al.^33^ the model reported here integrates cutting-edge CNNs to amplify the model’s generative capabilities and elevate the quality of the generated samples. CNNs have already orchestrated remarkable advancements in generative tasks and have showcased immense potential within the sphere of game optimization and data analysis. Through the incorporation of CNNs, this study’s model begets game optimization solutions and data analysis outcomes that resonate more precisely with particular requisites.

## Conclusion

Due to continuous advancements in science and technology, AI has become integral to driving societal progress. ECE, being a pivotal component of the contemporary educational landscape, faces the significant challenge of leveraging AI to further its development, thereby contributing to the overall growth of the education sector. Consequently, this study endeavors to enhance the quality of ECE by optimizing the GIADA model through the application of DL and CNN technology. The study commences by analyzing the current status of ECE within the DL framework and delves into fundamental principles of ECE game design. The research findings reveal that the DL-based model achieves data analysis accuracy ranging from 46 to 66%, 44 to 68%, 42 to 66%, and 46 to 64% across four datasets. It is evident that the basic model falls short in precisely analyzing data. Therefore, the DL-based GIADA model undergoes optimization using CNN to address this limitation. In this context, the DL-CNN GIADA model demonstrates exceptional data analysis accuracy, ranging from 83 to 93%, 82 to 93%, 81 to 93%, and 83 to 91% in the four datasets, respectively. The CNN technology proves highly effective in optimizing data analysis within the DL-based model. This study serves as a valuable resource for enhancing the framework of game design in the ECE major. While this study successfully constructs a robust technical model and conducts comprehensive evaluations, it is worth noting that further research is required to explore model integration and development. Subsequently, advocating for the utilization and expansion of AI in ECE remains an essential area for future investigation.

### Supplementary Information

Supplementary Figures.

## Data Availability

All data generated or analysed during this study are included in this published article [and its supplementary information files].
